# Expression of paralogous *SEP*-, *FUL*-, *AG*- and *STK*-like MADS-box genes in wild-type and peloric *Phalaenopsis* flowers

**DOI:** 10.3389/fpls.2014.00076

**Published:** 2014-03-12

**Authors:** Roberta Acri-Nunes-Miranda, Mariana Mondragón-Palomino

**Affiliations:** Department of Cell Biology and Plant Biochemistry, University of RegensburgRegensburg, Germany

**Keywords:** Orchidaceae, flower evolution, evo-devo, qPCR, gene duplication, labellum

## Abstract

The diverse flowers of Orchidaceae are the result of several major morphological transitions, among them the most studied is the differentiation of the inner median tepal into the labellum, a perianth organ key in pollinator attraction. Type A peloria lacking stamens and with ectopic labella in place of inner lateral tepals are useful for testing models on the genes specifying these organs by comparing their patterns of expression between wild-type and peloric flowers. Previous studies focused on *DEFICIENS-* and *GLOBOSA*-like MADS-box genes because of their conserved role in perianth and stamen development. The “orchid code” model summarizes this work and shows in Orchidaceae there are four paralogous lineages of *DEFICIENS/AP3*-like genes differentially expressed in each floral whorl. Experimental tests of this model showed the conserved, higher expression of genes from two specific *DEF*-like gene lineages is associated with labellum development. The present study tests whether eight MADS-box candidate *SEP-, FUL-, AG-*, and *STK*-like genes have been specifically duplicated in the Orchidaceae and are also differentially expressed in association with the distinct flower organs of *Phalaenopsis* hyb. “Athens.” The gene trees indicate orchid-specific duplications. In a way analogous to what is observed in labellum-specific *DEF*-like genes, a two-fold increase in the expression of *SEP3*-like gene *PhaMADS7* was measured in the labellum-like inner lateral tepals of peloric flowers. The overlap between *SEP3*-like and *DEF*-like genes suggests both are associated with labellum specification and similar positional cues determine their domains of expression. In contrast, the uniform messenger levels of *FUL-like* genes suggest they are involved in the development of all organs and their expression in the ovary suggests cell differentiation starts before pollination. As previously reported *AG*-like and *STK*-like genes are exclusively expressed in gynostemium and ovary, however no evidence for transcriptional divergence was found in the stage investigated. Gene expression suggests a developmental regulatory system based on the combined activity of duplicate MADS-box genes. We discuss its feasibility based on documented protein interactions and patterns of expression.

## Introduction

Over the last two decades comparative molecular genetic analysis of flower development and evolution have been fundamentally influenced by the ABC model of organ identity specification. This model resulted from the genetic analysis of floral homeotic mutants of *Arabidopsis thaliana* and *Antirrhinum majus* (Coen and Meyerowitz, 1991; Weigel and Meyerowitz, 1994).The original ABC and the extended ABCDE model associate the developmental determination of specific flower organs of *Arabidopsis thaliana* with the combinatorial activity of several classes of homeotic selector genes, most of which encode MADS domain developmental transcription factors: A- and E-class genes specify sepals; genes from classes A, B, and E determine petals; the combination of B-, C-, and E-class genes specify stamens; genes from class C and E determine carpels; and D- and E-class genes determine ovules (Reviewed in Theissen, 2001; Krizek and Fletcher, 2005).

Comparative studies demonstrate the conservation of homologs of the ABCDE genes across most Angiosperms (Becker and Theissen, 2003; Litt and Irish, 2003; Kramer et al., 2004; Zahn et al., 2005a,b, 2006), and suggest the regulatory principles of some of these homologs have been conserved during flower evolution (Whipple et al., 2004, 2007; Melzer et al., 2009; Cui et al., 2010).

The phylogenetic relationships of MADS-box genes have been investigated in depth and several studies consistently demonstrated *AP1-*/*FUL-*, *DEF-*/*GLO-*, *AG*-/*STK*-, and *SEP*-like genes form distinct phylogenetic groups (Purugganan et al., 1995; Theissen et al., 1996; Alvarez-Buylla et al., 2000; Becker and Theissen, 2003; Martinez-Castilla and Alvarez-Buylla, 2003; Nam et al., 2003). The finding that the genes in each clade or group of the MADS-box gene tree share distinct molecular sequences and have similar patterns of expression and functions suggests gene duplication and functional diversification of MADS-box genes played an important role in the evolution of flower morphology (Purugganan et al., 1995; Theissen et al., 1996). Furthermore, this relationship provides a solid comparative framework to generate and test hypotheses on the developmental program of non-model species.

It has been frequently observed that most non-grass monocot species like *Tulipa gesneriana*, *Agapanthus praecox* and *Muscari armeniacum* have several copies of *DEF*- and *GLO*-like genes that are expressed in the petaloid first whorl (Van Tunen et al., 1993; Kanno et al., 2003; Nakamura et al., 2005; Nakada et al., 2006). This shift in the pattern of expression respective to homologous genes from *Arabidopsis thaliana* and *Antirrhinum majus* is the basis of a proposed modification to the ABC model of flower identity specification for non-grass monocots. In this model *DEF*- and *GLO*-like genes determine tepal identity in the first whorl in addition to specifying inner tepal and stamen identity (Kanno et al., 2007). Nevertheless direct evidence supporting the modified ABC is still missing largely due to the technical hurdles of genetic analysis and stable transformation of non-grass monocots.

**Figure 1:**
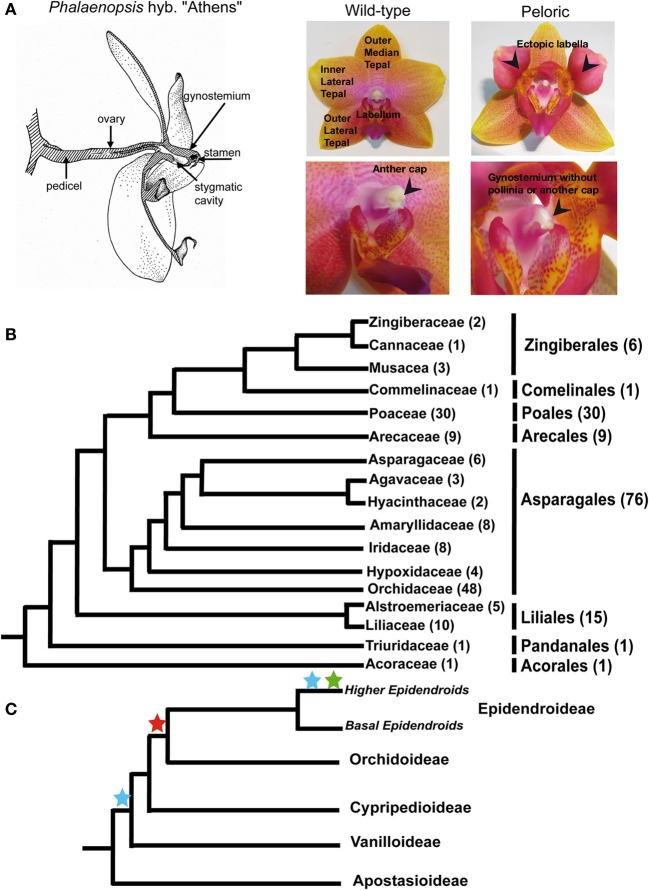
***Phalaenopsis* flower structure, distinctive floral features of wild-type and peloric hybrid “Athens.”** Phylogenetic relationships the sequences studied. **(A)** Flower organs of wild-type and peloric mutant represented in the analysis. The stamen is located under the anther cap, a white laminar structure on top of the wild-type gynostemium (indicated with an arrow). The labella developing in place of inner lateral tepals as well as the organs missing in the gynostemium of peloric flowers are indicated with arrows. **(B)** Systematic relationships of the plant families represented in the phylogenetic analyses of MADS-box genes from monocots (based on Angiosperm phylogeny website version 12, www.mobot.org). The number sequences from every group in the dataset is indicated between brackets. **(C)** Systematic relationships of Orchidaceae subfamilies. The stars mark the points where the Orchidaceae subfamily composition of the gene trees suggests duplications might have occurred in *SEP3*-like genes (pale and dark blue), *AG*-like genes (red) and *FUL*-like II (green). The colors of the stars correspond to those employed to indicate these duplications in Figures 2–4.

However, not all monocot species with petaloid flowers develop two whorls of identical perianth organs (tepals). Most flowers of Orchidaceae, the largest monocot family, have a highly differentiated zygomorphic perianth, including three types of organs: three outer tepals (sepals) in the first floral whorl, two inner lateral tepals (petals) as well as a frequently highly modified inner median tepal called labellum (or lip) in the second floral whorl (Rudall and Bateman, 2002) (Figure 1A). The labellum is positionally homologous to the adaxial tepal of other monocot flowers, but its position in the perianth is often the lowest because of a 180° torsion of the pedicel and/or ovary (resupination) that changes floral orientation (Arditti, 2002; Bateman and Rudall, 2006). The orientation of the labellum, its location in direct opposition to the fertile anther, its often distinct pattern of coloration as well as the presence of calli, spur, oil, or scent glands suggest its morphology is influenced by co-evolution with pollinators.

The previously described features of the orchid perianth, together with the partial to complete suppression of three to five of the original six stamens are some of the major morphological transitions in the floral evolution of Orchidaceae (Bateman and Rudall, 2006). The developmental origins of these transitions, especially the ontogeny of the labellum and its role in orchid reproduction have been recurring topics in botany and evolutionary biology since the 19th century. The finding that MADS-box class B genes specify perianth and stamen identity in *Arabidopsis* put forward *DEFICIENS*- and *GLOBOSA*-like genes as candidates for experimentally addressing some of the key morphological transitions in orchid flower evolution (Mondragón-Palomino, 2013).

Several studies showed that in Orchidaceae there are four ancient lineages of *DEF*-like genes (Tsai et al., 2004; Mondragón-Palomino and Theißen, 2008, 2009). Their phylogeny served as a point of reference to compare the expression of *DEF*-like genes in wild-type representatives of most orchid subfamilies and flower terata. This analysis yielded the “orchid code,” a model where differential combinatorial expression of *DEF*-like genes from specific clades is associated with the development of each type of perianth organ: outer tepals, inner lateral tepals and labellum (Mondragón-Palomino and Theißen, 2008, 2011; Mondragón-Palomino, 2013). Namely, specification of labellum identity is associated with higher levels of mRNA from both clade three (*PeMADS3*-like) and clade four genes (*PeMADS4*-like) in this organ (Mondragón-Palomino and Theißen, 2011).

The ABCDE model already indicates several genes contribute to the specification of the identity of each flower organ. The involvement of several factors in flower development reflects the fact that MADS-domain transcription factors form higher order complexes (Egea-Cortines et al., 1999) that bind to CArG-box motifs in the regulatory regions of their targets. According to the floral quartet model (Honma and Goto, 2001; Theissen and Saedler, 2001), in *Arabidopsis* tetramers formed by AP1 and SEP3, determine sepal development, AP1, PI, AP3, and SEP are involved in the specification of petals while complexes of AP3, PI, SEP, and AG determine stamen identity and tetramers formed by AG and SEP dimers control carpel development.

Therefore, in order to realistically approach orchid flower development it is necessary to see beyond the “orchid code” and integrate information on the number and patterns of expression of additional candidate regulators of flower development. This information would enable approaching the development and evolution of gynostemium (colum) and ovary, organs that significantly contribute to the morphological diversity of the family. The orchid gynostemium is formed by the complete or partial union of male and female organs. This structure is often employed as diagnostic character in orchid systematics because of its highly complex species-specific combination of appendages as well as the position and characteristics of pollinia and anthers (Dressler, 1993; Rudall and Bateman, 2002) (Figure 1A).

The orchid ovary is inferior with respect to the bases of the perianth organs and formed by three carpels. In most orchids there are no divisions between carpels, but in genera from subfamilies Apostasioideae and Cypripedioideae the ovary has three locules (Dressler, 1993). Investigating MADS-box candidate *SEP*-, *FUL*-, *AG*-, and *STK*-like genes would also contribute to understanding the development of the carpel before and after pollination, an event that in Orchidaceae triggers ovary development.

Previous studies reported the expression of *SEP*-, *FUL*-, *AG-*, and *STK-*like genes in orchids (Reviewed in Mondragón-Palomino, 2013). According to this work the evolution of these genes is characterized by several instances of gene duplication as well as a conserved pattern of expression of each duplicate gene. Specifically, *FRUITFULL*-like genes are expressed mostly in the gynostemium and in some instances also in the perianth (Yu and Goh, 2000; Skipper et al., 2005; Chen et al., 2007; Chang et al., 2009) while *AGAMOUS*- and *SEEDSTICK*-like genes are expressed in gynostemium and ovary (Song et al., 2006; Xu et al., 2006; Hsu et al., 2010; Wang et al., 2011; Chen et al., 2012; Salemme et al., 2013). Most of the *SEPALLATA*-like genes isolated so far are expressed in all flower organs (Lu et al., 1993; Yu and Goh, 2000; Johansen and Frederiksen, 2002; Yu et al., 2002; Xu et al., 2006; Chang et al., 2009).

In the present study we isolated eight *SEP*-, *FUL*-, *AG*-, and *STK-*like genes from *Phalaenopsis* hyb. “Athens,” investigated their phylogenetic and orthology relationships and compared the patterns of expression in the perianth, column and developing organs of wild-type and peloric flowers with labella in place of inner lateral tepals and neither pollinia nor anther cap (Figure 1A). The ectopic labella are regarded as such because their shape, size, thickness, color and presence of calli are identical to those of the wild-type labellum.

The aims of this work are to investigate the association of additional MADS-box genes with the development of the labellum and pollinia, determine if paralogous *SEP*-, *FUL*-, *AG*-, and *STK-*like genes are also differentially expressed in the organs and stages investigated and in doing so contribute, in a way analogous to *DEF*-like genes, to the modularization of the orchid perianth. Although expression of some of the genes here investigated has been previously measured, not all studies include additional paralogs nor involve the simultaneous comparison of several MADS-box genes in homeotic organs or the developing ovary.

The results suggest a system of flower organ identity specification based on duplicate genes some of which are differentially expressed. We discuss the possible occurrence of dosage effects and their role in the preservation of these ancient duplicates. Because of the high prevalence of gene and genome duplication in plants, the origin and transcriptional divergence of orchid duplicate MADS-box genes reflect important processes shaping flower development, evolution and diversification in Angiosperms.

## Materials and methods

### Plant material

The origin and growth conditions of *Phalaenopsis* hybrid “Athens” (Epidendroideae) with wild-type or peloric flowers were described in Mondragón-Palomino and Theißen (2011). The organs of wild-type and peloric flower buds from of 0.9 to 1.0 cm in length were dissected, shock-frozen with liquid N_2_ and stored at −80°C.

### RNA isolation and cDNA synthesis

Frozen flower organs and developing ovaries were individually ground with sterile steel beads. Total RNA was extracted with a buffer containing 8 M Guanidin-HCl, 20 mM MES, 20 mM EDTA pH 7,0. After a phenol, isoamyl alcohol, chloroform (25:24:1) extraction and centrifugation RNA was precipitated overnight with 1 N acetic acid and 100% ethanol. After centrifugation at 15,000 rpm for 10 min pellets were washed in 80% ethanol, dried by centrifugation and resuspended in 30–70 μ l RNase-free, sterile water. All steps were carried at 4°C.

Genomic DNA was removed with DNase I (RNase-free) from Fermentas (1 U/μ l, Fermentas, http://www.fermentas.com), following the manufacturer's protocol. Concentration, integrity and quality of total RNA were measured on an Agilent 2100 Bioanalyzer (Agilent Technologies, http://www.genomics.agilent.com).

Synthesis of cDNA was performed with 1 μ g of total DNAse-treated RNA from each sample with oligo (dT)_15_ AB05 as previously described (Mondragón-Palomino and Theißen, 2011). The quality of the resulting cDNA was verified by assessing the amplification of the internal reference genes *Actin*, *Ubiquitin* and *EF1α* of *Phalaenopsis* on an 1% agarose gel.

### Primer design

All primer pairs were designed with Primer3 v. 0.4.0 (http://frodo.wi.mit.edu/primer3). The sequences employed as target for primer design were identified in ML clade-specific alignments and phylogenies of orchid *FUL*-, *AG*-, *STK*-, and *SEP*-like genes previously described. The specificity of each primer pair was verified by the size of its amplicon in a 1% agarose gel. Annealing temperatures were obtained by gradient PCR with 1:5 diluted cDNA from flower buds or developing ovaries as template. The reactions were performed as described by the manufacturer (http://fermentas.com) in a Biometra TProfessional basic thermocycler gradient (http://www.biometra.de). Amplification efficiency (E) for each primer pair was calculated as previously described (Mondragón-Palomino and Theißen, 2011). *Actin*, *Ubiquitin*, and *EF1α* of *Phalaenopsis* were amplified with the corresponding primers from Mondragón-Palomino and Theißen (2011). All primers were ordered from biomers.net and their sequences are available in Supplementary Table I.

### Quantitative real-time RT-PCR assays

Assays were performed with Peqlab KAPA SYBR FAST qPCR Master Mix Universal (http://www.peqlab.com), in a Real-Time Thermo Cycler Realplex (Eppendorf, http://www.eppendorf.de). For each target gene and organ triplicate reactions with cDNA from each of two independent cDNA pools were performed. Samples were arranged in a 96-well plate according to the principle of maximization (Derveaux et al., 2010). In each plate were included a positive control (cDNA from flower buds), a non-template negative control (NTC) and two samples of DNase treated total RNA pooled from wild-type and peloric flowers to detect any genomic DNA contamination.

The quality of qPCR assay was assured by: sequencing the resulting products (Supplementary Figure 1), detection of a single amplicon of predicted size in a 1% agarose gel; a single specific peak in the melting curve of triplicate reactions, the cycle threshold (Cq) value of samples within a triplicate should not deviate by more than 0.50 cycle as well as on the validation of positive and negative controls. We employed the amplification conditions described in (Mondragón-Palomino and Theißen, 2011).

### Normalization and data analysis

The Cq values of individual qPCR runs were exported to qBase plus v. 1.5 (Biogazelle, http://www.biogazelle.com) for further analysis. This program implements a modified ΔΔ *Ct* method (Hellemans et al., 2007) that takes the gene-specific amplification efficiencies calculated for each primer pair with standard curves. In this case, qBase plus employed the Cq values of *EF1α*, *Ubiquitin*, and *Actin* to generate a normalization factor. Normalization against three or more validated reference genes is considered most appropriate and universally applicable method (Vandesompele et al., 2002) and enables comparison with previous analysis of *DEF*- and *GLO*-like MADS-box gene expression in wild-type and peloric *Phalaenopsis* “Athens” (Mondragón-Palomino and Theißen, 2011).

The normalized quantities were rescaled relative to the sample with the lowest relative quantity, together with the corresponding standard errors they were exported to Excel v. 12.2.7 for Mac (Microsoft, http://www.microsoft.com) and to R v. 2.3.1 (R Foundation for Statistical Computing, http://www.r-projetic.org/foundation) to assess the correlation between samples from the same clade with Spearman's correlation test.

### Sequences and molecular phylogenies

Keyword and BLAST searches in public databases (Supplementary Table I) with known MADS-box *SEP*-, *FUL*-, *AG*-, and *STK-*like genes from *Zea mays* and *Oryza sativa* returned 48 candidate orchid MADS-box sequences. To determine their evolutionary relationships and gene duplication history we performed computational alignments and inferred their relationships to other non-grass monocots. To support clade definition we included sequences of MADS-box genes from *Oryza sativa* and *Zea mays* (Supplementary Table II).

Sequences were obtained from across monocots, including Asparagales (76), Poales (30), and Liliales (15) (Figure 1B). The family best represented is Orchidaceae with 48 sequences, mostly from *Phalaenopsis*, *Dendrobium*, *Oncidium*, and *Cymbidium*, species of horticultural interest from the Epidendoideae, the largest and most derived orchid subfamily (Figure 1C).

Automated sequence alignment was carried out using the program Muscle v. 3.8.31 (Edgar, 2004) implemented in SeaView v. 4.3.5 (Gouy et al., 2010). All alignments were visually checked, manually improved and employed to obtain the corresponding nucleotide alignments.

Molecular phylogenies were inferred with Maximum Likelihood (ML) implemented in SeaView v. 4.3.5. ML analysis were performed with an optimized BioNJ tree as starting topology, a GTR model of substitution, aLRT (SH-like) as branch support, optimized invariables sites, optimized across-site rate variation. Tree searching operations took the best result from Nearest-Neighbor-Interchange and Subtree-Pruning-and-Regrafting.

The gene tree of *FUL*-like genes was rooted with a sample of basal angiosperm *FUL*-like genes which were previously employed for similar analyses (Pabón-Mora et al., 2013). *SEP-like* genes were rooted with a sample of *SQUA*-like genes from Angiosperms. In order to increase the resolution of both major clades *SEP3*- and *SEP1*, *2*, and *4*-like genes from basal Angiosperms were included. *AG*- and *STK*-like genes were rooted with Gymnosperms *AG*-like sequences following the example of (Kramer et al., 2004). All sequences employed for rooting and improving ingroup resolution are listed in Supplementary Table II.

## Results

### Orchidaceae-specific duplications characterize the phylogenies of *SEP-, FUL-, AG-*, and *STK-*like genes

**Figure 2:**
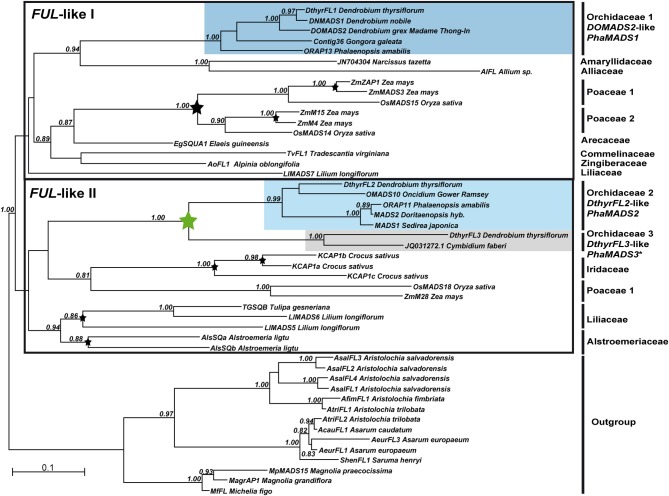
**Maximum Likelihood phylogeny of monocot *FUL*-like genes**. The two major monocot clades previously identified are indicated with a black frame (Litt and Irish, 2003). The clades containing sequences from Orchidaceae are highlighted if gene expression was characterized in *Phalaenopsis* hyb. “Athens.” Otherwise the non-isolated ortholog is marked with an asterisk. The numbers on every node indicate branch support above 0.80. The bars indicate clades of *FUL*-like genes from the plant families represented in the dataset as well as the outgroup. Black arrows point at the sequences employed as templates for qPCR primer design. Stars indicate gene duplications.

**Figure 3:**
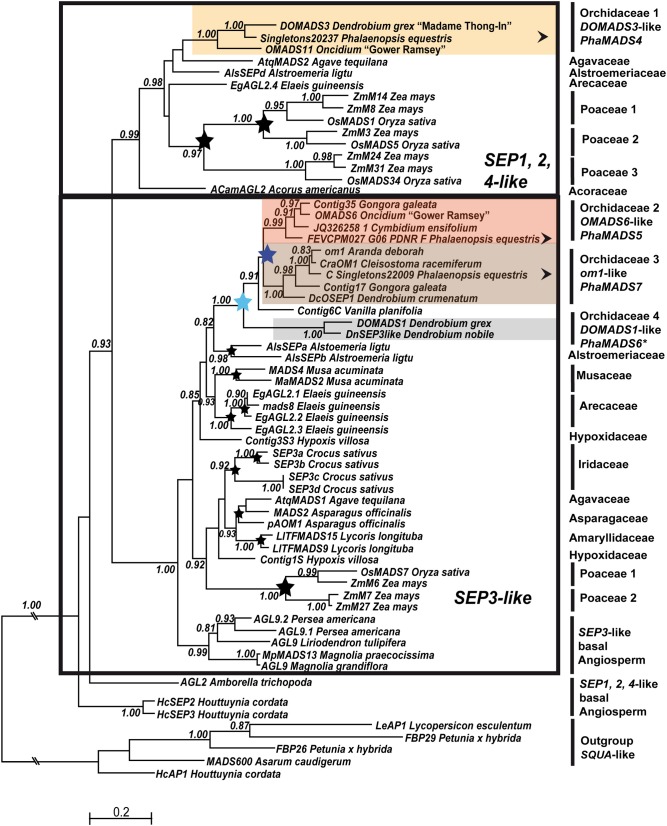
**Maximum Likelihood phylogeny of monocot *SEP*-like genes**. The two major monocot clades previously identified are indicated with a black frame (Zahn et al., 2005a). Orchidaceae-specific clades and other annotations are as described in Figure 2.

**Figure 4:**
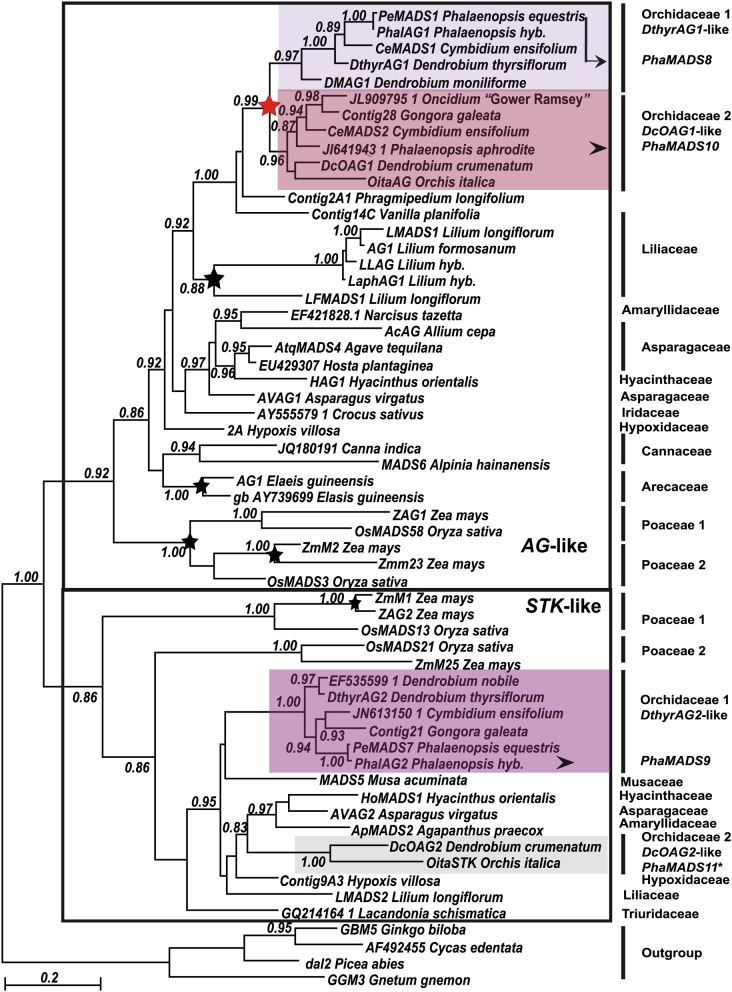
**Maximum Likelihood phylogeny of monocot *AG*- and *STK*-like genes**. Major monocot clades previously identified are indicated with a black frame (Kramer et al., 2004). Orchidaceae-specific clades and other annotations are as described in Figure 2.

Overall, the majority of the sequences from the same plant family form clades supported with probabilities larger than 0.95 (Figures 2–4). Although the phylogenetic relationships among these clades are topologically similar to those of their corresponding plant families (Figures 1B,C), they are often supported with probabilities smaller than 0.90.

Our phylogenetic analysis of monocot *APETALA1*/*FRUITFULL* genes identified the two major groups *FUL*-like I and *FUL*-like II previously inferred by Litt and Irish (2003). Despite employing a broader sample of monocot sequences, these groups are still unresolved as their low statistical support suggests. Notwithstanding, in both groups our analysis confirmed several grass- and species-specific gene duplications in *Crocus*, *Lilium*, and *Alstroemeria*. Furthermore in the *FUL*-like I subgroup there is a single, clade of orchid-specific *DOMADS2*-like genes clearly grouped with other genes belonging to species in Asparagales. In the *FUL*-like II genes there are two well-supported clades of *DthyrFL2*- and *DthyrFL3*-like sequences. However their relationship to other genes from species in Asparagales is not well-resolved (Figure 2).

While the C-terminal domain encoded by *DthyrFL1* and *DthyrFL2*-like genes has the conserved *FUL*-like motif LPPWML, the sequences encoding *DthyrFL3*-like proteins have an early stop codon that eliminates the motif (Supplementary Figure 2A). As suggested by the alignment, the loss of this motif has already taken place in the common ancestor of *Dendrobium* and *Cymbidium* (Epidendroideae).

Similar changes in the region encoding the C-terminal domain caused by early stop codons and major deletions leading to loss of regions potentially involved in protein-protein interactions have been documented in *OMADS3*-like *DEF*-like genes form Orchidaceae (Mondragón-Palomino et al., 2009). The divergence of the C-terminal domains of both *OMADS3-* and *DthyrFL3*-like genes might imply their proteins adopted novel patterns of higher order molecular interaction (Supplementary Figure 2A) (Mondragón-Palomino et al., 2009).

In comparison with proteins like ZAP1 and ZmMADS3, which have C-terminal domains rich in glutamine and serine, the corresponding orchid FUL-like II proteins do not have similar long homopolymeric stretches. Because some of these repetitive sequences have been involved in transcriptional activation and associated to morphological evolution (Gerber et al., 1994; Lindqvist et al., 2007) their absence in orchid FUL-like proteins suggests divergence of their functional properties.

The analysis of *SEP*-like genes supports a division in two major clades: *SEP1*, *2*, *4*-like genes and *SEP3*-like genes. In monocots *SEP1*, *2*, *4*-like genes are characterized by four subclades, three of them [*OsMADS1*, *OsMADS5*, und *RMADS217*-like genes (*OsMADS34*)] are grass-specific (Zahn et al., 2005a) (Figure 3). In the single, non-resolved group containing non-grass species we identified a well-supported group of *DOMADS3-like* genes (Figure 3). Like other monocot lineages, the C-terminal domain of *DOMADS3*-like genes has a conserved SEP I motif, but also a rather divergent or missing SEP II motif (Supplementary Figure 2C).

In the analyses of (Zahn et al., 2005a), monocot *SEP3*-like genes are divided in three major groups, two of them being grass-specific. In the non-grass sequences we reproducibly identified several well-supported species-specific duplications in *Alstroemeria*, *Musa*, *Eleais*, *Crocus*, *Asparagus*, and *Lycoris* (Figure 3). Nonetheless, the node at the base of non-grass monocot group is supported with a probability lower than 0.8.

In Orchidaceae we identified two successive family-specific duplications (Figure 3). The earliest one involves the clade of *DOMADS1*-like genes and a group containing *Vanilla planifolia* Contig6C and the ancestor of *OMADS6*- and *om1*-like genes, which subsequently resulted from a second duplication event. The sequences in *OMADS6*- and *om1*-like genes have well-conserved SEP I and SEP II motifs while both *DOMADS1*-like genes have a divergent K-domain and a truncated C-domain (Supplementary Figure 2C). However, sequences from a broader sample of species are needed to precisely date the origin of these clades and the process behind their diversification.

In the case of monocot *AG*-like MADS-box genes, previous analyses reported three clades, two of them exclusively involving Poaceae-specific sequences and duplications (Kramer et al., 2004). The present analysis shows that in addition to the grass subclades there is a group of non-grass genes involving several species-specific duplications in Liliaceae and Arecaceae. Although the relationships among most non-grass sequences are statistically well supported, they do not always reproduce those of the plant groups where they belong.

Our analysis identified two clades of Orchidaceae-specific *AG*-like genes: *DthyrAG1*-like and *DcOAG1*-like (Figure 4). The relationship of both clades to sequences from *Phragmipedium longifolium* (Cypripedioideae) and *Vanilla planifolia* (Vanilloideae), two relatively basal orchid species (Figure 1B), suggests these clades might be the result of a relatively recent duplication in Epidendroideae or already in Orchidoideae (Figure 4). Furthermore, the analysis supports the relationship between the Orchidaceae clade and that of Liliaceae *AG*-like genes.

Previous analyses of monocot *STK*-like genes identified two sister clades of Poaceae-specific genes (Kramer et al., 2004). Outside of these groups the present analysis identified a poorly resolved group of non-grass genes. Among them, there are two clades of *DthyrAG2*- and *DcOAG2-*like genes from Orchidaceae (Figure 3). Although the relationship between both clades with each other and the rest of the sequences is not clear, they share conserved AGI and AGII motifs (Supplementary Figure 2B) and several Orchidaceae-specific substitutions, indels and in the case of *DcOAG2*, an early stop codon that eliminates the last seven amino acids of its C-terminal domain.

### Patterns of expression

#### FUL-like genes PhaMADS1 and PhaMADS2 are highly expressed in the ovary before pollination

Both *FUL*-like I *PhaMADS1* and *FUL*-like II *PhaMADS2* are transcribed at a relatively uniform and low level in perianth and gynostemium and are expressed at their highest level in the ovary before pollination.

**Figure 5:**
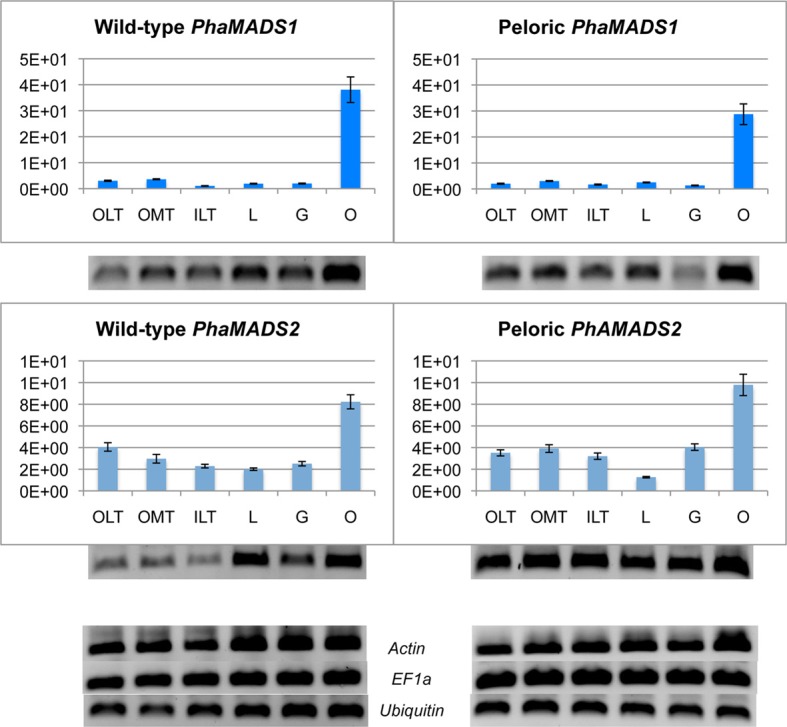
**Normalized expression of orchid *FUL*-like genes**. Expression of *PhaMADS1* and *PhaMADS2* in six flower organs of wild-type and peloric of *Phalaenopsis* hybrid “Athens.” OLT, outer lateral tepals; OMT, outer median tepals; ILT, inner lateral tepals; L, labellum; G, gynostemium; O, ovary. Expression of the target genes was normalized to the geometric average expression of three internal control genes: *Actin*, *EF1α* and *Ubiquitin*. Each column represents the expression obtained from six samples (three replicates from each of two different cDNA pools). The error bars represent the standard errors of the replicates. The y-axis is in arbitrary fluorescence units. The qPCR products from each sample series are presented below their corresponding columns in the graph.

The similarity in the patterns of expression of these paralogs in wild-type and peloric flowers is reflected by a correlation of 0.88 (Spearman's test) (Figure 5). At the level of individual organs a noteworthy difference is the 60% increase on the expression of *PhaMAD2* in the gynostemium of peloric flowers (Figure 5). In addition, a low level of expression of *PhaMADS1*, but especially of *PhaMADS2* was detected in leaves (Supplementary Figure 3).

In this study it was not possible to isolate the *Phalaenopsis* ortholog of *DthyrFL3*, representative of the second clade of *FUL*-like II genes (Figure 2). Because previous studies have not documented the expression of genes from this lineage in individual flower organs, it is not clear whether in addition to being expressed in developing ovules they are also associated with perianth formation (Skipper et al., 2005).

#### SEPALLATA-like gene PhaMADS7 is differentially expressed in inner lateral tepals and labellum

**Figure 6:**
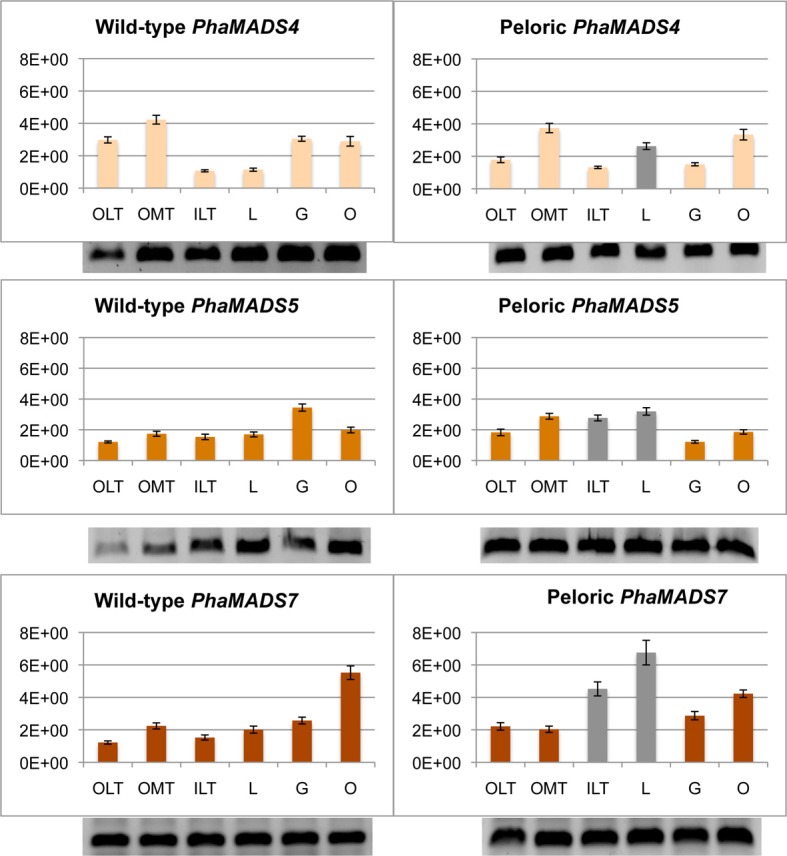
**Normalized expression of orchid *SEPALLATA*-like genes**. Expression of *PhaMADS4* and *PhaMADS5* and *PhaMADS7* in six flower organs of wild-type and peloric of *Phalaenopsis* hybrid “Athens.” Replicates, normalization, graphics and abbreviations are as in Figure 5.

*SEP*-like genes *PhaMADS4*, *PhaMADS5*, and *PhaMADS7* are expressed in all flower organs. Among the eight genes here measured the *SEP3*-like gene *PhaMADS7* showed the largest expression differences between wild-type and peloric flowers: in the labellum-like inner lateral tepals its expression increased 196% and in the labellum 235% (Figure 6). In contrast, in the outer lateral tepals its expression increased 81%. The expression of *PhaMADS5*, the second *SEP3*-like gene measured, also increased in the outer median tepal, inner lateral tepals and labellum of peloric flowers, albeit at a lower level (65, 80, and 88% respectively) and decreased by 65% in the peloric gynostemium (Figure 6). The expression of *SEP1*-, *2*, *4*-like gene *PhaMADS4* also showed an interesting increased of 130% in the peloric labellum (Figure 6).

Furthermore, messengers of *PhaMADS5* were detected in wild-type leaves and roots as well as in leaves of plants producing peloric flowers, while expression of *PheMADS7* was detected in wild-type leaves (Supplementary Figure 3).

#### AGAMOUS-like genes PhaMADS8 and PhaMADS10 and STK-like gene PhaMADS9 are only expressed in gynostemium and ovary

**Figure 7:**
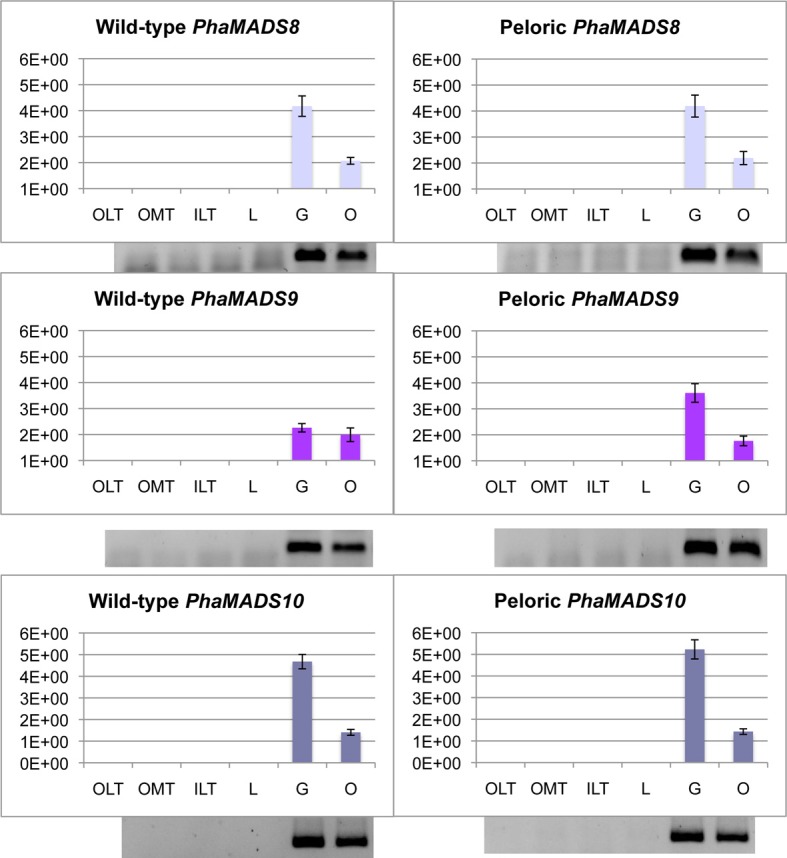
**Normalized expression of orchid *AGAMOUS*- and *SEEDSTICK*-like genes**. Expression of *PhaMADS8*, *PhaMADS9* and *PhaMADS10* in six flower organs of wild-type and peloric *Phalaenopsis* hybrid “Athens.” Replicates, normalization, graphics and abbreviations are as described in Figure 5.

In gynostemium and ovary there are relevant differences on the relative level of messengers from *PhaMADS8* and *PhaMADS9* (both *AG*-like genes), and *PhaMADS10* (*STK*-like gene). While *PhaMADS8* and *PhaMADS10* are expressed twice as high in the gynostemium as in the ovary there is no significant difference in the expression of *PhaMADS9* in those organs (Figure 7).

*PhaMADS9* is expressed 60% more in the peloric gynostemium as in the wild-type (Figure 7). In contrast, there are no significant changes in the levels of expression of *PhaMADS8* and *PhaMADS10* in the organs of wild-type and peloric flowers. Messengers for none of these three genes were detected in leaves and roots (Supplementary Figure 3).

#### Differential activity of FUL-, AG-, STK-, and SEP-like genes during ovary development in phalaenopsis

**Figure 8:**
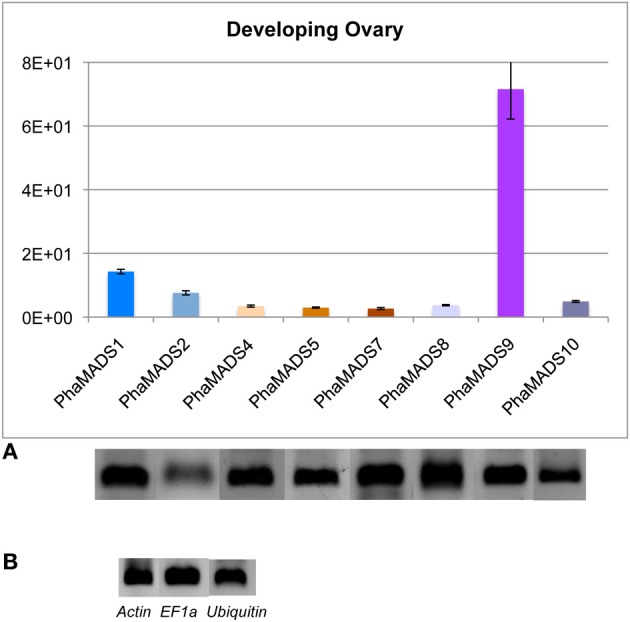
**Normalized expression of orchid *FUL*-, *SEP*-, *AG*-, and *STK*-like MADS-box genes in developing ovary. (A)** Normalized expression of MADS-box genes *PhaMADS1*, *PhaMADS2* (*FUL*-like), *PhaMADS4*, *PhaMADS5*, *PhaMADS7* (*SEP*-like), *PhaMADS8*, *PhaMADS10* (AG-like) and *PhaMADS9* (*STK*-like) in developing ovary of *Phalaenopsis* hybrid “Athens.” **(B)** Expression of three internal control genes (*Actin*, *EF1α* and *Ubiquitin*) in the developing ovaries. Replicates, normalization, graphics and abbreviations are as described in Figure 5.

Expression of the eight *SEP*-, *FUL*-, *AG*-, and *STK*-like genes previously described was also measured in the developing ovary of *Phalaenopsis* hyb. 56 days after pollination (DAP) (Supplementary Figure 4), when ovule differentiation takes place after a phase of ovary growth and proliferation of ovule primordia (Zhang and O'neill, 1993). The significant differences we observed mostly involve the *STK-*like gene *PhaMADS9*, which expressed 5–27 times higher than the rest of the transcripts measured (Figure 8). The levels of expression in the ovary before and after pollination cannot be compared because they were measured in tissues of a different *Phalaenopsis* variety due to ovary abortion after self- or cross-pollination of *Phalaenopsis* hyb. “Athens.”

## Discussion

### Orchidaceae duplicate MADS-box genes have been retained for millions of years

Because of the high representation of Orchidaceae genes in the phylogenies, we identified several well-supported, family-specific duplications which broaden the previously identified monocot MADS-box groups *FRUITFUL*- (*FUL-*), *AGAMOUS*- (*AG-*), *SEEDSTICK*- (*STK-*), and *SEPALLATA*-like (*SEP-*) genes, (Litt and Irish, 2003; Kramer et al., 2004; Zahn et al., 2005a, 2006). Because the sample of MADS-box genes from Orchidaceae species is rather biased toward subfamily Epidendroideae the conclusions drawn from this dataset regarding number of paralogs and date of duplication might turn out to be different as a broader dataset becomes available.

The phylogenetic relationships identified within the clades of *FUL*-like *DOMADS2*-like genes and *DtyhrFL2*-like genes are consistent with previous phylogenetic analyses (Chen et al., 2007). Because genes from Orchidaceae are present in both *FUL*-like I and *FUL*-like II monocot clades our analysis suggests these groups originated earlier in Angiosperm evolution than initially dated (Litt and Irish, 2003). The phylogenetic analysis here presented reproduces recent studies involving Orchidaceae *AG*-/*STK*- (Salemme et al., 2013) and describes the occurrence of two duplications in Orchidaceae *SEP3*-like genes.

The groups of duplicate Orchidaceae *SEP*-, *FUL*-, *AG*-, and *STK*-like genes are ancient as suggested by their origin at different points in the history of Orchidaceae (Figure 1C). The molecular phylogenies suggest the duplication generating *OMADS6*- and *om1*-like *SEP3* genes (Figure 3) probably took place after the divergence of family Vanilloideae, at least 62 million years ago (MYA) (Ramirez et al., 2007). In contrast, the duplication of *DthyrAG1*- and *DcOAG1*-like *AG* genes is relatively more recent, probably involving the ancestor of subfamily Orchidoideae, about 56 MYA (Figure 4). Genes in all other clades are already present in several species from the so-called “higher” Epidendroids, whose origin dates back to 54 MYA (Ramirez et al., 2007). As more data becomes available, it will become clearer whether these apparently recent paralogs are Epidendroideae-specific or more ancient (Figure 1C).

The mechanisms behind the retention of these paralogs are possibly associated with dosage effects. Assuming the different levels of messengers result in distinct amounts of protein products, it is foreseeable their concentration plays a role in the formation of regulatory protein complexes and eventually the activation of target genes. In this context, the biased retention of *Phalaenopsis* duplicate MADS-box genes is feasibly explained by the gene balance hypothesis, which states duplicated gene retention following a Whole Genome Duplication (WGD) would avoid the harmful consequences of dosage imbalance among interacting proteins. Dosage effects are already implied by the association of different levels of *DEF*-like genes *PeMADS3* and *PeMADS4* and the development of inner lateral tepals and labellum (Mondragón-Palomino and Theißen, 2011).

### Expression of *SEP-, FUL-, AG-*, and *STK*-like genes in *Phalaenopsis*

The two-fold increase of *PhaMADS7* (*SEP3*-like) expression in the inner lateral tepal and the labellum of peloric flowers suggests it might have a distinct role in the development of the inner perianth. These aspects will be discussed in “A transcriptional model for *Phalaenopsis* flower development.”

Our results agree with previous work reporting *SEP*-like genes from orchids are expressed in all flower organs during development, in a way analogous to *SEP1*, *SEP2*, and *SEP3* from *Arabidopsis thaliana* (Lu et al., 1993; Yu and Goh, 2000; Johansen and Frederiksen, 2002; Yu et al., 2002; Xu et al., 2006; Chang et al., 2009) (Summarized in Supplementary Table III). Based on these studies it has been argued that in orchids *SEP*-like genes are involved in floral transition and flower organ identity specification.

Our findings in *Phalaenopsis* also agree with the fact in other non-Poales monocots like *Asparagus officinalis* (dioecious, Asparagaceae), *Eleais guineensis* (monoecious Arecaceae) and *Musa acuminata* (dioecious, *Musaceae*) *SEP*-like genes are expressed in the inflorescence, in all flower organs and flower meristems and thus might be involved in their development (Caporali et al., 2000; Tzeng et al., 2003; Adam et al., 2007; Tsaftaris et al., 2011) as well as in the differences between male and female flowers of *Musa acuminata* (Elitzur et al., 2010) and *Agave tequilana* (Agavaceae) (Delgado Sandoval et al., 2011).

The domains of expression of *FUL*-like genes *PhaMADS1* and *PhaMADS2* were generally similar to those of their orthologs in other orchid species (Yu and Goh, 2000; Skipper et al., 2005; Chen et al., 2007; Chang et al., 2009) (summarized in Supplementary Table IIIA). However they differ with previous studies in that both genes are also expressed in the perianth organs (Figure 5). This might be due to the fact gene expression was measured in developing tepals where distinctive features like shape, color and appendages are not yet defined while *DOMADS2* and *OMADS10*, *ORAP11*, and *ORAP13* were measured in fully-developed tepals (Yu and Goh, 2000; Chen et al., 2007; Chang et al., 2009) (Summarized in Supplementary Table III).

The relatively high level of expression of *PhaMADS1* and *PhaMADS2* in the ovary agrees with previous studies suggesting that monocot *FUL* genes might have a role fruit development in a way analogous to *Arabidopsis*' *FRUITFULL* rather than to *AP1* (Litt and Irish, 2003). In accordance to this, the sequences of *PhaMADS1* and *PhaMADS2* like all members of the *AP1*/*FUL*, *AGL6*, and *SEP* gene subfamilies share a conserved, C-terminal, hydrophobic *FUL*-like and the M/LPPGWLA SEPII motives (Supplementary Figures 2A,B respectively) (Litt and Irish, 2003; Zahn et al., 2005a).

In *Arabidopsis FRUITFULL* is key to fruit morphogenesis after fertilization by mediating elongation and cell differentiation within fruit valve layers (Gu et al., 1998). Because orchid ovary development starts after fertilization, it is intriguing that *PhaMADS1* and *PhaMADS2* are highly expressed before this event, thus implying unknown aspects of ovary cell differentiation might have an earlier starting point (Figure 5).

Although sequence similarity with *FUL* does not explain the expression of both genes in all four whorls, it is a characteristic shared with other non-grass monocots like: *Crocus* (Iridaceae), *Tradescantia* (Commelinaceae), *Lilium* (Liliaceae), *Agapanthus* (Agapanthaceae), and *Elaeis* (Arecaceae) (Tsaftaris et al., 2004; Preston and Kellogg, 2006; Adam et al., 2007). In our view the patterns observed support the notion that in non-grass monocots expression of *FUL*-like genes in all four floral whorls is the ancestral state (Preston and Kellogg, 2006) and suggests in these species *FUL*-like genes might also play a role in the development of all flower organs. Nonetheless a genetic definition of class A function in non-grass monocots would require analysis of mutants or specific silencing of each paralog.

The expression of *PhaMADS8*, *PhaMADS10* (both *AG*-like) and *PhaMADS9* (*STK*-like) in gynostemium and ovary agrees with most previous transcriptional characterizations of *AG*- and *STK*-like genes in orchids (Skipper et al., 2006; Song et al., 2006; Xu et al., 2006; Hsu et al., 2010; Wang et al., 2011; Chen et al., 2012; Salemme et al., 2013) (Summarized in Supplementary Table III). Exceptionally, and probably due to differences in the developmental stages analyzed, messengers for *AG*-like genes *DcOAG1*, *CeMADS2*, and *OitaAG* as well as *STK*-like gene *OitaSTK* are also detected in perianth organs (Xu et al., 2006; Wang et al., 2011), particularly in stages closer to anthesis (Salemme et al., 2013).

The highly similar patterns of expression of duplicate *AG*- and *STK*-like genes suggest they are redundantly involved in the development of gynostemium and ovary in the stages investigated. Nevertheless recent studies on *multitepal* (*Cymbidium ensifolium)* and *glyp* (*Phalaenopsis*) might suggest otherwise. In *multitepal* the gynostemium is replaced by an ectopic flower which produces outer and inner tepal-like structures centripetally (Wang et al., 2011), in a way analogous to *agamous* from *A. thaliana*. In the wild-type, *AG*-like genes *CeMADS1* (ortholog of *PhaMADS8*) and *CeMADS2* (ortholog of *PhaMADS10*) are strongly expressed in the gynostemium while *CeMADS2* is weakly expressed in the perianth organs. However, *CeMADS1* is not expressed in the gynostemiumless buds of the *multitepal* mutant and *CeMADS2* remains weakly expressed in the perianth (Wang et al., 2011). Further analysis is needed to determine whether the level of expression reported supports a role in perianth development.

In agreement with our results *PeMADS1* (ortholog of *PhaMADS8*) and *PeMADS7* (ortholog of *PhaMADS9*) are expressed in ovary and gynostemium of *Phalaenopsis equestris* (Chen et al., 2012). However they might play different roles in the development of these structures as suggested by the differential expression of *PeMADS1* in the gynostemium-like inner lateral tepals of the *glyp* mutant of *Phalaenopsis* hyb. “CD1” (Chen et al., 2012).

Further analysis of gynostemium and ovary mutants is required to discern the specific roles in column development of paralogous orchid *AG-* and *STK*-like genes.

In agreement previously described work in orchids, *AG*-like genes in other non-grass monocots, like *LLAG1*, *HAG1*, *AcAG*, *CsAG1a*, and *AtqMADS4* from *Lilium longiflorum*, *Hyacinthus orientalis*, *Allium cepa*, *Crocus sativus*, and *Agave tequilana* respectively, are expressed in stamens and carpels, suggesting a role in the development of these organs by analogy to the domains of expression of *AGAMOUS* in the ABC model (Li et al., 2002; Benedito et al., 2004; Tsaftaris et al., 2005; Hsu et al., 2010; Delgado Sandoval et al., 2011; Li et al., 2013).

In contrast to the expression of orchid *STK*-like genes in gynostemium and ovaries (Skipper et al., 2006; Song et al., 2006; Xu et al., 2006; Hsu et al., 2010; Chen et al., 2012; Salemme et al., 2013) (Supplementary Table III), non-grass monocots genes *LMADS2* (*Lilium longiflorum*), *HoMADS1* (*Hyacinthus orientalis*), and *LsSTK* (*Lacandonia schismatica*) are expressed exclusively in the carpel, mainly in the ovules (Tzeng, 2002; Xu et al., 2004; Alvarez-Buylla et al., 2010). In Orchidaceae expression in the gynostemium is probably due to adnation of stamens and style.

### A transcriptional model for *Phalaenopsis* flower development

The classic ABC model of flower organ identity specification was based on genetic analysis of single and multiple mutants affecting distinct groups of flower organs in *Antirhinum majus* and *Arabidopsis thaliana*. Although initially the genes behind the phenotypes observed were not known, it was possible to employ the tools of classical genetics to understand how combinations of different functions affected the development of specific organs in particular whorls (Bowman et al., 1991).

Although a similar approach is not yet feasible in orchids, an initial, informative approach is associating the patterns of expression of *DEF*-, *GLO*- *SEP*-, *FUL*-, *AG*-, and *STK-*like MADS-box genes in wild-type and mutant flowers with the development of particular organs. The resulting patterns are the basis of models on evolution and development that later on can be genetically evaluated.

**Figure 9:**
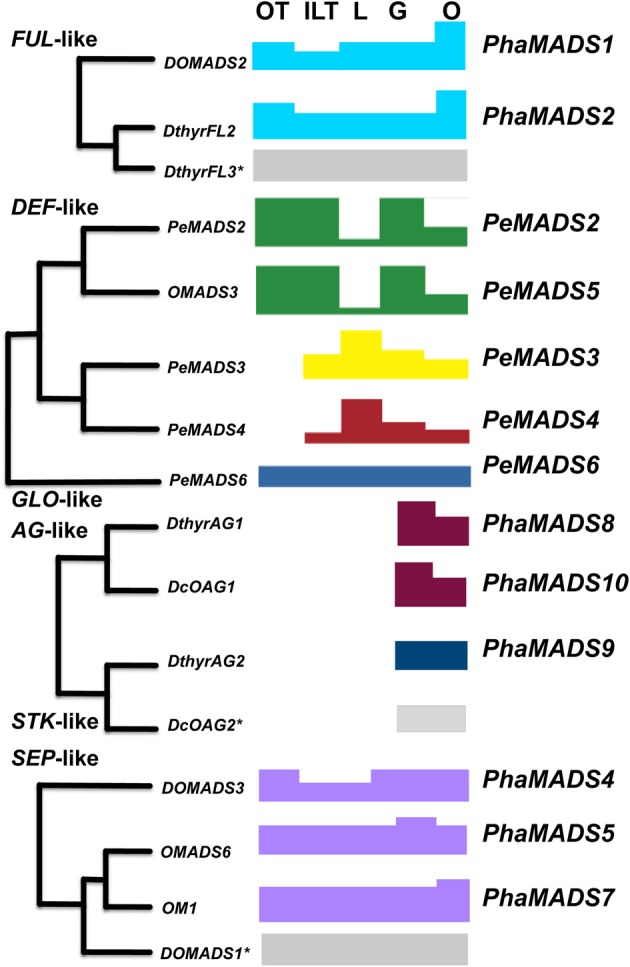
**A transcriptional model for *Phalaenopsis* flower development**. Summary of expression patterns of orchid paralogous *FUL*-, *DEF*-, *GLO*-, *AG*-, *STK*-, and *SEP*-like genes in *Phalaenopsis* perianth and reproductive organs. The combined differential activity and levels of expression of genes from different clades in each organ are represented with blocks of different colors and mapped on their domains of expression in the perianth, column and ovary. Genes corresponding to a clade with an asterisk were isolated and measured in other studies and their pattern of expression is included here for completion.

In the following paragraphs we propose how the genes profiled would be involved in the specification of distinct flower organ identities in *Phalaenopsis* (Summarized in Figure 9).

#### Outer tepal identity: DEF-, GLO-, SEP-, and FUL-like genes

In this system of organ identity specification outer tepals would be defined by the differential expression of *DEF*-like genes *PeMADS2* (clade 1) and *PeMADS5* (clade 2) together with *PeMADS6* (*GLO*-like gene) (Mondragón-Palomino and Theißen, 2011) (Figure 9). *SEP*-like genes would contribute to the specification of this whorl as they do in the case of *Arabidopsis thaliana* and rice (Pelaz et al., 2000; Cui et al., 2010). Because of its higher level of expression we assume *PhaMADS4* (*SEP1, 2, 4*-like) makes a larger contribution to organ identity determination in this whorl (Figures 6, 9).

The activity of the three *FUL*-like genes identified might be relevant for the development of the outer perianth. However, a floral mutant affected on the first whorl must first be analyzed to determine their specific role.

#### Inner lateral tepals and labellum identity is associated with differential activity of specific DEF- and *SEP3*-like genes

The increase in expression of *SEP3*-like gene *PhaMADS7* in the peloric inner perianth suggests an association between its differential expression (Figure 6) and the development of the inner lateral tepals and labellum. Most importantly, the highest levels of expression of this gene in peloric flowers overlap with those of *PeMADS3* and *PeMADS4*, two *DEF*-like genes, which in a similar way are highly expressed in peloric inner-lateral tepals and wild-type labellum and therefore have been associated to the development of this organ (Mondragón-Palomino and Theißen, 2011) (Figure 9). Because the pattern of differential expression of *SEP3*-like gene *PhaMADS7* increased in the peloric inner perianth in a way analogous to that of *PeMADS3* and *PeMADS4* it is feasible their domains of expression are determined by a common upstream regulatory gene whose activity reflects adaxial-abaxial positional cues on the flower meristem.

The feasibility of regulatory protein-protein interactions between the transcription factors encoded by *PhaMADS7* (*SEP3*-like) and both *DEF*- and *GLO*-like genes has been experimentally documented in orchid *Dendrobium crumenatum* between DcOAP3B, DcOPI, and DcOSEP1, product of the ortholog of *PhaMADS7*. These complexes are analogous to those formed in *Arabidopsis thaliana* by class B proteins AP3 and PI with SEP3, their most abundant interaction partner (Smaczniak et al., 2012). These proteins together with AP1 or AG are key in the specification of petal and stamen identity, respectively (Honma and Goto, 2001; Theissen and Saedler, 2001).

#### Stamen development in the gynostemium

In *Arabidopsis thaliana* MADS-box gene classes B, C, and E determine the development of stamens. Our results suggest the abortion of stamen development might be associated to the important change of expression of *PeMADS2* (*DEF*-like, clade 2) in the peloric gynostemium (Mondragón-Palomino and Theißen, 2011) as well as the 65% decrease in the expression of *SEP3*-like gene *PhaMADS5* (Figure 6), which encodes a potential interaction partner for PeMADS2. In contrast, there are not changes in the expression of *AG*-like genes *PhaMADS8* and *PhaMADS10* (Figure 7).

#### Ovary and ovule development: SEP-, FUL-, AG-, and STK-like genes

The functional conservation of the genes controlling ovary and ovule development in eudicots and monocots (Favaro et al., 2002, 2003; Dreni et al., 2007; Cui et al., 2010), suggests PhaMADS9 (STK-like), encoded by the gene most highly expressed in the ovary after pollination, might play a role in ovule identity determination, by forming complexes with the products of co-expressed *PhaMADS8* and *PhaMADS10* (both *AG*-like) as well as *PhaMADS4*, *PhaMADS5* and *PhaMADS7* (*SEP*-like) genes (Figure 9).

## Conclusions and perspectives

Clearly the dataset presently available for phylogenetic reconstruction of MADS-box gene evolution in non-grass monocots must still be broadened beyond the species represented in this study to enable detailed inference of the number and age of paralogs in each group of genes as well as comparative analysis of molecular orchid evo-devo.

In this context, our study in *Phalaenopsis* hyb. “Athens” suggests, albeit a few exceptions, that the MADS-box genes investigated generally share their patterns of expression with other non-grass monocots. A key difference however, is that for a fraction of Orchidaceae, and especially for *Phalaenopsis* it is already clear each group of genes is represented by two or more paralogs (Figures 2–4, 9). The joint expression of these paralogs hints at a system of regulatory activities determined by the differential expression of some of them in specific domains (Figure 9).

Concretely, the profiles obtained suggests flower organ identity results from the activity of multiple groups of duplicate *FUL*-, *DEF*-, *AG*-, *STK*-, and *SEP*-like genes. Orchid flower morphology might then be to a great extent the result of an extended developmental “toolkit” that in the course of evolution enabled a complex network of regulatory interactions with a broader, organ-specific group of downstream targets. Previous analysis of the patterns of expression in orchid *DEF*-like MADS-box genes suggested the subfunctionalization of developmental paralogs might increase the genotypic modularity of organisms by generating novel domains of expression (Mondragón-Palomino and Theißen, 2008). Once duplicate genes acquired different domains and levels of expression, they also might attain particular groups of interaction partners and downstream targets. The resulting developmental modules (e.g., the labellum or the gynostemium) might then respond independently to natural selection (e.g., pollinators) eventually giving rise to morphologically distinct flower organs.

The prevalent occurrence of gene duplication has major consequences for the regulation of orchid flower development and requires addressing the following topics:

(a) ***Positional cues defining gene expression***

While *FUL*, *SEP*-, and *GLO*-like genes seem to be expressed in all flower organs, *DEF*-, AG-, and STK-like genes have domains of expression in specific perianth or reproductive organs (Figure 9). This difference suggests the expression of certain genes is sensitive to specific positional cues. Understanding their nature and mechanisms of action is key to learning how duplicates diverged transcriptionally and became associated to the development of particular organs.

(b) ***Functional redundancy***

In order to clarify whether *AG*-, *FUL*-, and *SEP3*-like paralogs are transcriptionally redundant it would be necessary to investigate a broader array of developmental stages and tissues with approaches like RNA-seq on microdissected tissues. On the other hand, classic genetic analysis or genetic transformation (transient or stable) of at least one from the recently advanced model species (reviewed in Mondragón-Palomino, 2013) would offer a more direct approach to their actual function or functions.

(c) ***Role of C-terminal domain variation in protein-protein interactions***

In Orchidaceae *FUL*-, *DEF*-, *AG*-, *STK*-, and *SEP*-like genes there is one clade where the sequence of the C-terminal domain substantially diverges from all others investigated (Supplementary Figure 2 and (Mondragón-Palomino et al., 2009)). It remains to be elucidated whether the proteins encoded by the members of these distinct lineages modify the formation of regulatory higher order interactions.

## Author contributions

Roberta Acri-Nunes-Miranda collected plant material, performed experiments, analyzed data, prepared illustrations and drafted the manuscript. Mariana Mondragón-Palomino designed the study, analyzed data, prepared illustrations and wrote manuscript.

### Conflict of interest statement

The authors declare that the research was conducted in the absence of any commercial or financial relationships that could be construed as a potential conflict of interest.

## Supplementary material

Supplementary Figure S1 **Sequences of qPCR amplicons**. Amino acid translation of the sequences used for qPCR primer design and the corresponding sequences of the products amplified with such primers. Due to sequencing the amplicon size is not identical to the one given in Supplementary Table II.

Supplementary Figure S2 **C-terminal domains of *FUL*-, *AG*/*STK* and *SEP*-like genes from Orchidaceae**. Sequences are grouped according to the clades in the corresponding phylogenies. Framed in black are conserved sequence motifs mentioned in the text. Color-coding indicates the biochemical properties of each amino acid.

Supplementary Figure S3 **Expression of orchid *FUL*-, *SEP*-, *AG*-, and *STK*-like MADS-box genes in leaves and roots. (A)** Expression of seven MADS-box genes in leaves (L) and roots (R) of *Phalaenopsis* hybrid “Athens” wild-type and peloric. C stands for control amplification with wild-type flower bud cDNA. **(B)** Expression of three internal control genes (*Actin*, *Ubiquitin*, and *EF1α*) in the cDNA pools from leaves and roots.

Supplementary Figure S4 **Transverse sections of developing ovary from *Phalaenospsis* hyb. 56 DAP. (A)** Distal section of monocarpellate ovary with parietal placentation. **(B)** Proximal section (near to the pedicel). P and O indicate placenta and ovules, respectively.

Supplementary Table I **Characteristics and sequences of the primers pairs employed for qPCR**.

Supplementary Table II **List of sequences employed in alignments, phylogenetic analysis and primer design**.

Supplementary Table III **Expression of *FUL*-, *AG*/*STK* and *SEP*-like MADS-box genes from Orchidaceae**.
