# Supplementary figures and images for: Expression of paralogous SEP-, FUL-, AG- and STK-like MADS-box genes in wild-type and peloric Phalaenopsis flowers
Source: Front Plant Sci. 2014 Mar 12;5:76. doi: 10.3389/fpls.2014.00076 (PMC3950491; doi:10.3389/fpls.2014.00076)

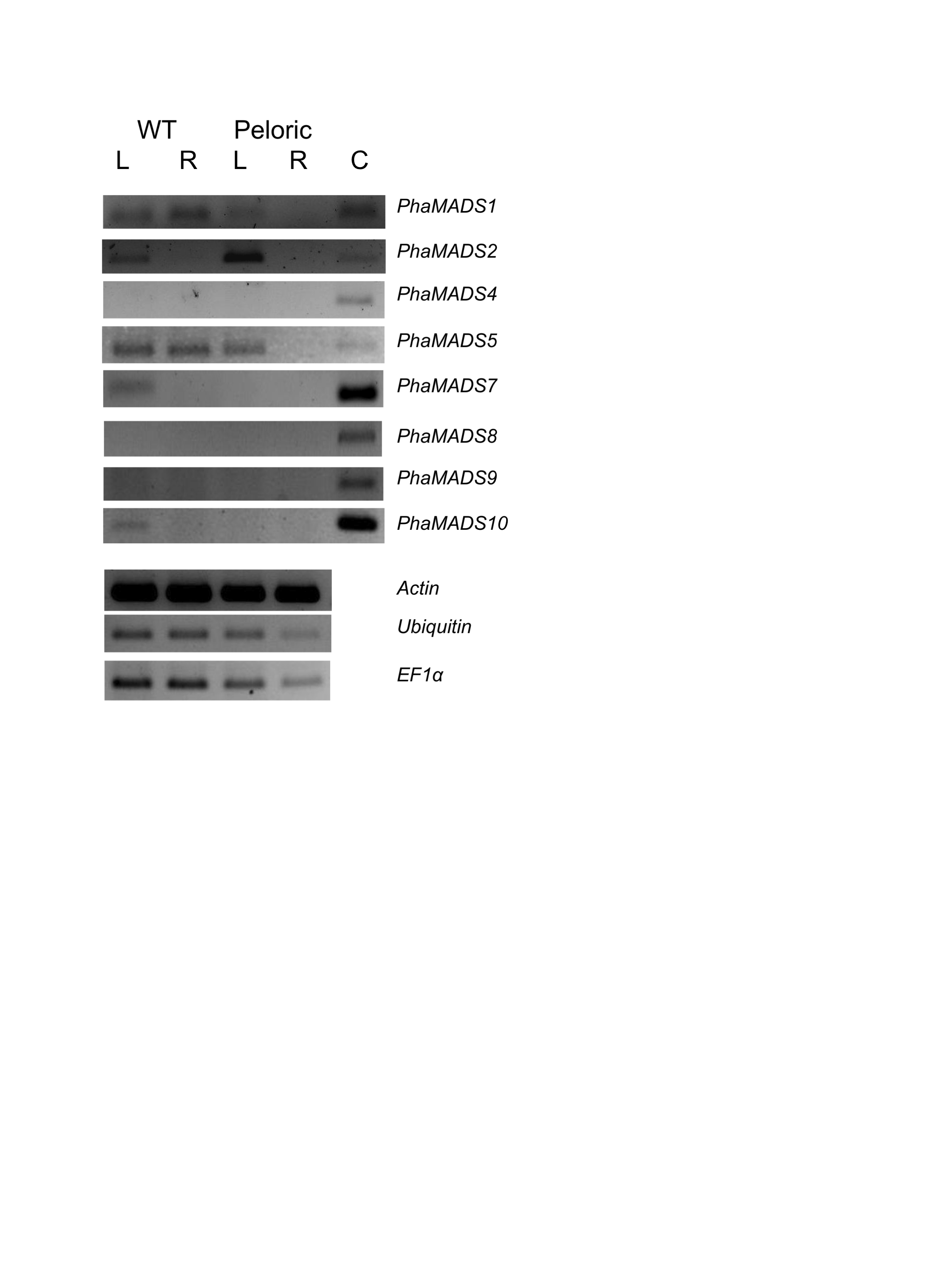

Supplement: Supplementary Figure S1 — Sequences of qPCR amplicons. Amino acid translation of the sequences used for qPCR primer design and the corresponding sequences of the products amplified with such primers. Due to sequencing the amplicon size is not identical to the one given in Supplementary Table II. [file DataSheet1.ZIP › 75310_Mondragón_Palomino_Suppl_Figure_3.TIF]

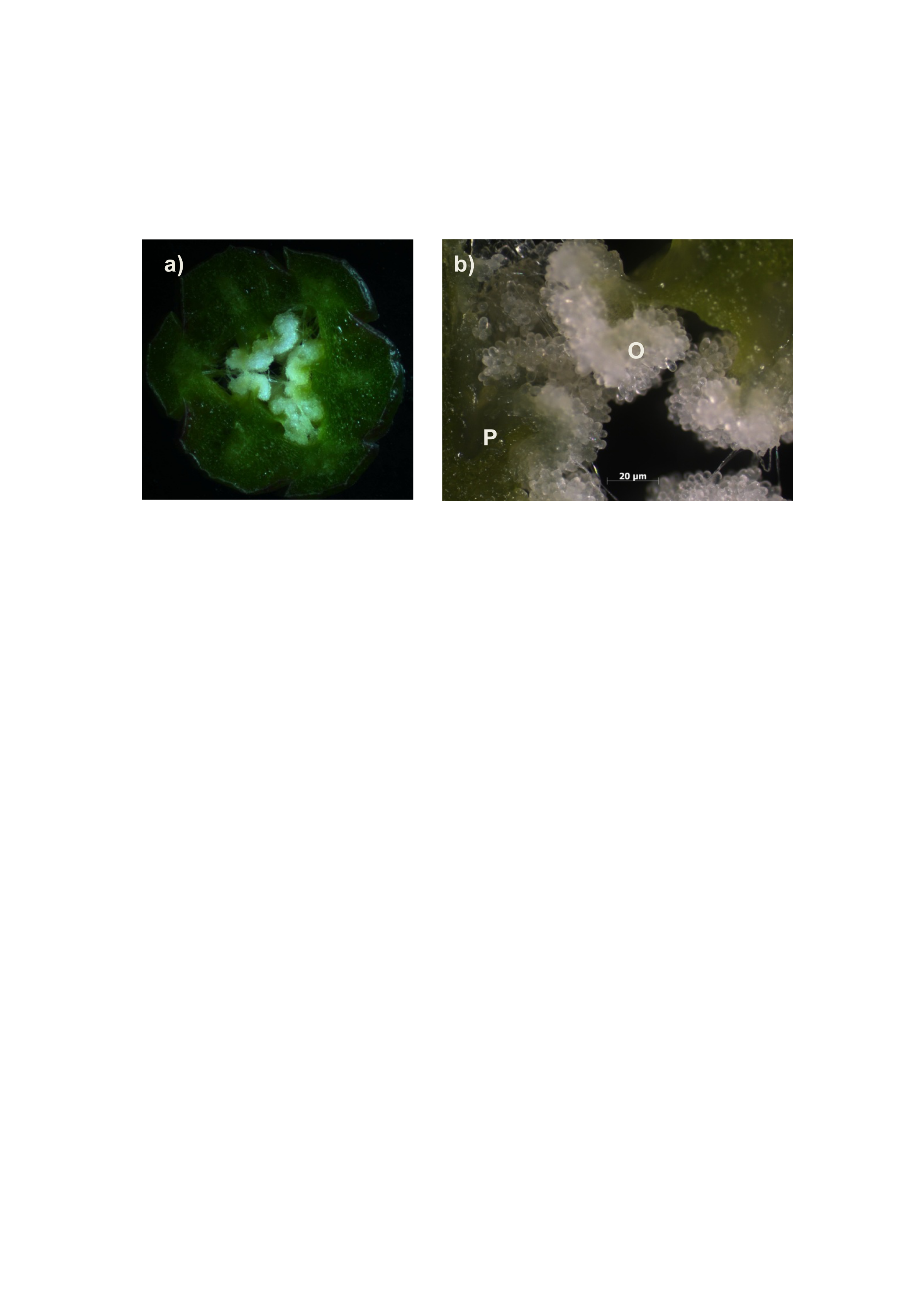

Supplement: Supplementary Figure S1 — Sequences of qPCR amplicons. Amino acid translation of the sequences used for qPCR primer design and the corresponding sequences of the products amplified with such primers. Due to sequencing the amplicon size is not identical to the one given in Supplementary Table II. [file DataSheet1.ZIP › 75310_Mondragón_Palomino_Suppl_Figure_4.TIF]

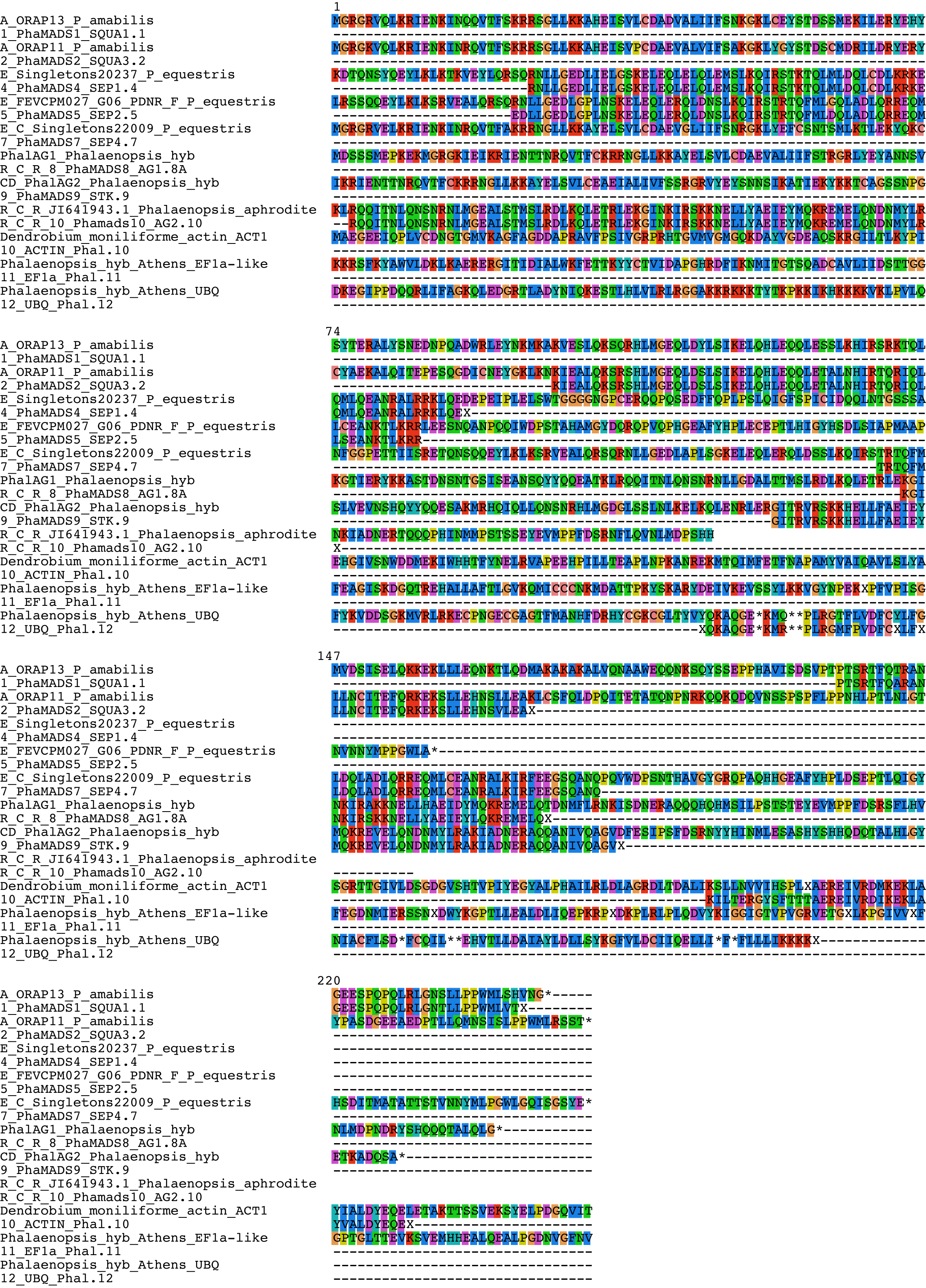

Supplement: Supplementary Figure S1 — Sequences of qPCR amplicons. Amino acid translation of the sequences used for qPCR primer design and the corresponding sequences of the products amplified with such primers. Due to sequencing the amplicon size is not identical to the one given in Supplementary Table II. [file DataSheet1.ZIP › 75310_Mondragón_Palomino_Suppl_Figure_1.TIF]

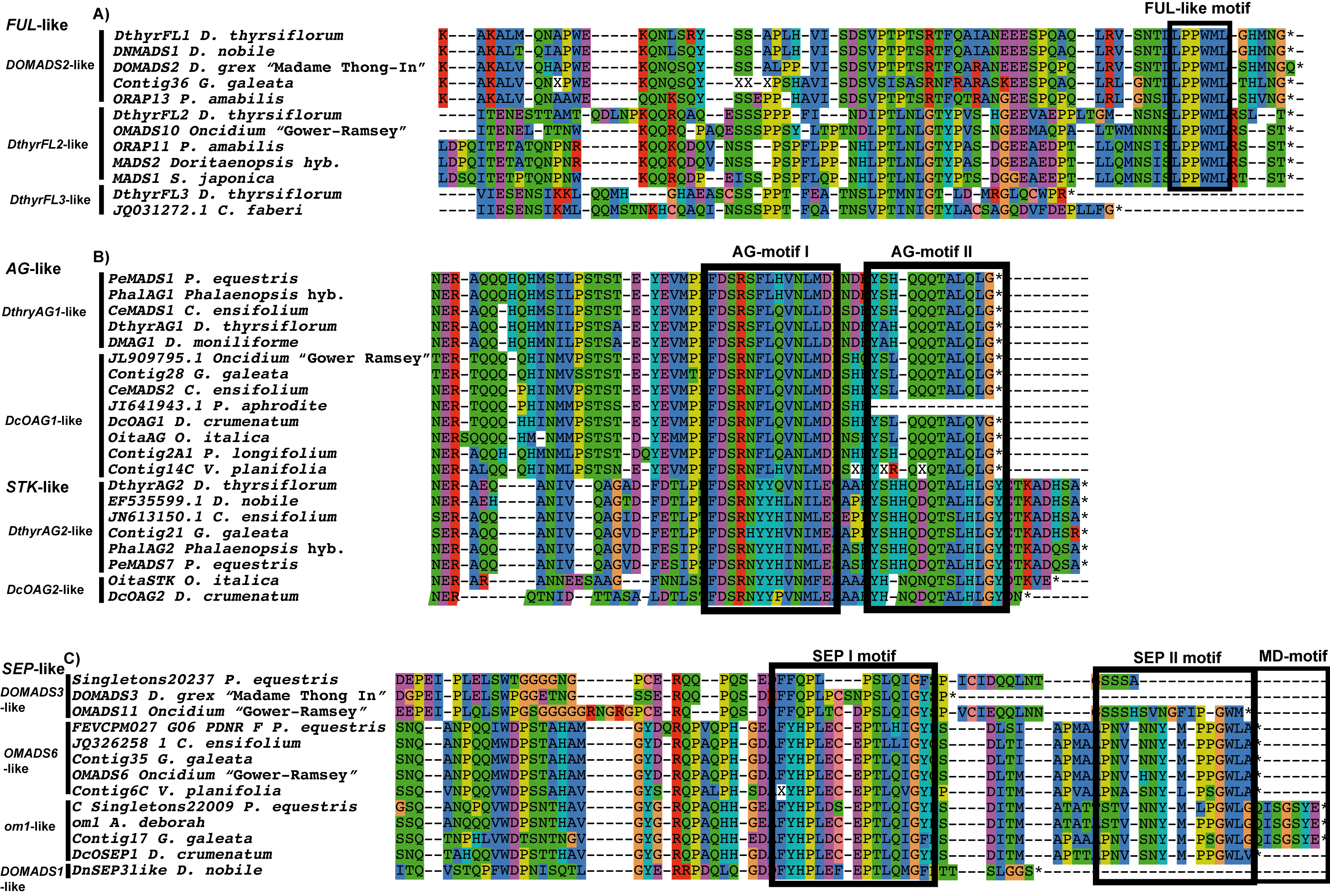

Supplement: Supplementary Figure S1 — Sequences of qPCR amplicons. Amino acid translation of the sequences used for qPCR primer design and the corresponding sequences of the products amplified with such primers. Due to sequencing the amplicon size is not identical to the one given in Supplementary Table II. [file DataSheet1.ZIP › 75310_Mondragón_Palomino_Suppl_Figure_2.TIF]
